# A case of isolated allergy to sheep's milk

**DOI:** 10.1186/2045-7022-3-S3-P157

**Published:** 2013-07-25

**Authors:** R Bonaguro, F Lazzarotto, A Toniolo, C Zorzin, MA Muraro

**Affiliations:** 1 Department of Pediatrics - University of Padua, Food Allergy Centre - Veneto Region, Padova, Italy

## Background

A 6 year old boy came to our attention reporting two recent episodes of diffuse urticaria and eyelid and lip swelling, accompanied by a burning feeling of the oral mucosa and tingling of the throat. In the first episode symptoms appeared after ingestion of a dessert homemade by his grandma: a layer of dough (wheat flour, water and lard) filled with soft cheese and lemon juice, fried in peanut oil and covered by honey. In the second episode, again at his grandma’s, symptoms had a more rapid onset, after the very first bite of a compound made of cheese and parsley. From the age of 6 months the boy had had mild atopic dermatitis; at the age of 3 years he had undergone SPT which were positive for egg white, peanut and grass pollen. Since then he had been on a diet free of egg, peanut and tree nuts; he had recently started to eat baked products containing egg with no symptoms. The boy was commonly eating lemon juice, honey, parsley as well as cow’s milk and its derivates.

## Methods

**SPT** (standard reagents): positive for egg white, grass and tree pollens. **Prick to prick** with peanuts and tree nuts: negative. **Specific IgE testing:** peanut 4,86 kU/L (ara h 1 0,15 kU/L; ara h 2, 3 negative); egg white 0,5 kU/L; parsley 3,99 kU/L; cow’s milk 0,6 kU/L (αlactoalbumin 0,29; βlactoglobulin 0,12; casein 0,13); grass pollen >100 kU/L; birch pollen 2 kU/L. Since the clinical episodes could not be explained either by the patient’s history, nor by the result of the tests, a deeper questioning in the family was conducted and finally emerged that: 1) the grandma had used two different types of cheese – cow’s milk (CM) cheese and sheep’s milk (SM) cheese – to prepare both the filling of the dessert and the cheese-parsley compound ; 2) the boy had never had sheep cheese before; 3) sheep cheese was the only new food ingested by the boy in the two episodes.

## Results

**Prick to prick test** (wheal diameter in mm): sheep’s cheese= 12; fresh sheep’s milk= 8; fresh cow’s milk= negative; histamine= 7.

## Conclusion

The boy was allergic to SM and derivates, in the absence of CM allergy. A mild sensitization to CM proteins was present, but any related allergy, if ever, was clearly outgrown. The sensitization to egg white and parsley had no clinical relevance. Diagnosis could be possible only after further exploring the patient’s clinical history.

## Disclosure of interest

None declared.

